# Preoperative Repetitive Navigated TMS and Functional White Matter Tractography in a Bilingual Patient with a Brain Tumor in Wernike Area

**DOI:** 10.3390/brainsci11050557

**Published:** 2021-04-28

**Authors:** Valentina Baro, Samuel Caliri, Luca Sartori, Silvia Facchini, Brando Guarrera, Pietro Zangrossi, Mariagiulia Anglani, Luca Denaro, Domenico d’Avella, Florinda Ferreri, Andrea Landi

**Affiliations:** 1 Academic Neurosurgery, Department of Neuroscience, University of Padova, 35128 Padova, Italy; samucaliri91@gmail.com (S.C.); sartori.luca.92@gmail.com (L.S.); brandoguarrera@gmail.com (B.G.); pietro.zangrossi@gmail.com (P.Z.); luca.denaro@unipd.it (L.D.); domenico.davella@unipd.it (D.d.); andrea.landi@unipd.it (A.L.); 2 Department of Neuroscience DNS, University of Padova, 35128 Padova, Italy; facchini.silvia@gmail.com; 3 Unit of Neuroradiology, Padova University Hospital, 35128 Padova, Italy; mariagiulia.anglani@aopd.veneto.it; 4 Unit of Neurology and Neurophysiology, Department of Neuroscience, University of Padova, 35128 Padova, Italy; florinda.ferreri@unipd.it

**Keywords:** transcranial magnetic stimulation, brain tumor, bilingual, language, preoperative mapping, case report

## Abstract

Awake surgery and intraoperative neuromonitoring represent the gold standard for surgery of lesion located in language-eloquent areas of the dominant hemisphere, enabling the maximal safe resection while preserving language function. Nevertheless, this functional mapping is invasive; it can be executed only during surgery and in selected patients. Moreover, the number of neuro-oncological bilingual patients is constantly growing, and performing awake surgery in this group of patients can be difficult. In this scenario, the application of accurate, repeatable and non-invasive preoperative mapping procedures is needed, in order to define the anatomical distribution of both languages. Repetitive navigated transcranial magnetic stimulation (rnTMS) associated with functional subcortical fiber tracking (nTMS-based DTI-FT) represents a promising and comprehensive mapping tool to display language pathway and function reorganization in neurosurgical patients. Herein we report a case of a bilingual patient affected by brain tumor in the left temporal lobe, who underwent rnTMS mapping for both languages (Romanian and Italian), disclosing the true eloquence of the anterior part of the lesion in both tests. After surgery, language abilities were intact at follow-up in both languages. This case represents a preliminary application of nTMS-based DTI-FT in neurosurgery for brain tumor in eloquent areas in a bilingual patient.

## 1. Introduction

Surgical resection of lesions involving the language pathway remains a major challenge for the neurosurgeon, harboring a risk of new functional deficits. Repetitive navigated transcranial magnetic stimulation (rnTMS) has proven to provide a reliable non-invasive preoperatory cortical mapping for language function, showing a good overall correlation with intraoperative direct cortical stimulation (DCS) [1,2,3,4,5]. Nevertheless, its sensitivity, specificity, negative and positive predicting values varies widely among studies. Therefore, rnTMS speech mapping is the only method that can replace DCS when the latter cannot be performed [6,7,8,9]. Subcortical tracts can be identified by diffusion tensor imaging-fiber tracking (DTI-FT) based on rnTMS mapping, obtaining an accurate and functionally oriented white matter preoperative study. In fact, it allows planning of the best surgical strategy for resection, improving postoperative outcome, especially in patients who are not eligible for awake surgery [8,10,11,12,13,14,15]. A detailed preoperative mapping of the language pathway is mandatory, especially in case of bilingual patients, a peculiar subgroup that can present different patterns of cortical representation of the languages. In fact, the first language (L1) and the second language (L2) are processed both by shared brain areas as well as language-specific areas [16]. Moreover, even in L1 and L2 shared areas distinct language-specific neural population for the different languages have been identified by rnTMS [17]. Furthermore, Tussis et al. studied the cortical distribution of L1 and L2 in the non-dominant hemisphere with rnTMS, disclosing the involvement of dorsal precentral and middle precentral gyrus especially for L1, and triangular inferior frontal gyrus for L2 [18]. Whereby, a comprehensive preoperative understanding of the language pathway may be useful also in patients eligible for awake surgery, enabling a custom tailored craniotomy size and a faster and safer cortical mapping [2]. Herein we present the case of a 54-year-old Romanian woman affected by a primary brain tumor in the left angular gyrus who underwent preoperative rnTMS mapping to explore both Romanian and Italian languages. In the following, neurosurgical planning, surgical intervention and outcome are described and discussed.

## 2. Case Presentation

### 2.1. Patient Information, Clinical and Radiological Findings

**Figure 1:**
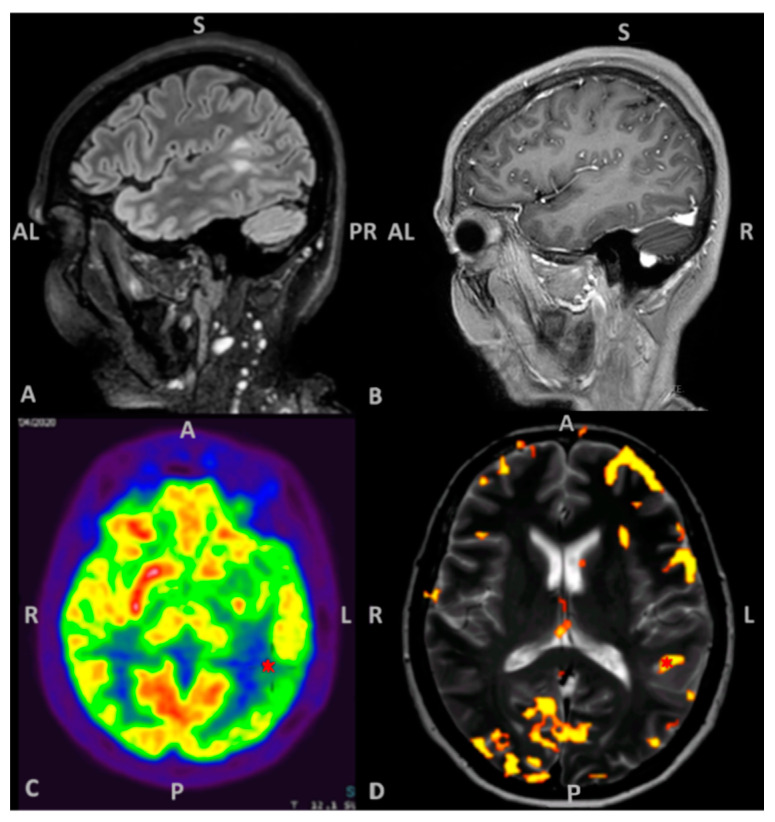
(**A**) 3D FLAIR (fluid attenuated inversion recovery) image discloses a primitive brain tumor located between the posterior part of the superior and middle temporal gyri and the anterior part of the angular gyrus in the left hemisphere; (**B**) the lesion does not enhance after contrast medium administration; (**C**) the 18F-fluorodeoxyglucose PET/MRI reveals the hypometabolism of the tumor (*). (**D**) Axial T2w image fused with the BOLD (blood oxygenation level dependent) signal activation map obtained during word generation task shows a focal cortical activation in the superior–anterior part of the lesion (*).

A right-handed, bilingual 54-year-old woman was admitted at the emergency department for a generalized tonic clonic seizure sustained by a primitive brain tumor located between the posterior part of the superior and middle temporal gyri and the anterior part of the angular gyrus in the left hemisphere. The lesion did not enhance after contrast medium administration and it was hypometabolic at 18F-fluorodeoxyglucose PET/MRI. The functional MRI (fMRI) confirmed that the lesion was located in the dominant hemisphere (Figure 1). Due to the anxiety of the patient, mostly related to the diagnosis of brain tumor, the fMRI was performed testing only her mother tongue, i.e., Romanian. Interictal EEG showed an irritative activity in left centro–parietal derivations.

### 2.2. Neuropsychological Evaluation

Concerning the social and work surrounding the patient had been living in Italy for 17 years with her family, perfectly integrated in the social context, working as a housekeeper. Previously, she had 13 years of education, graduating in a vocational school in her home country.

The patient underwent a comprehensive battery of standardized neuropsychological tests performed in Italian, in order to evaluate the impact of the tumor on cognitive functions. A standardized evaluation of Romanian language was not executable because native language versions of the tests were not available and because none of the team spoke Romanian. The assessment was composed of tests covering different cognitive domains. The Oxford Cognitive Screen [19,20], a brief screening instrument composed of tasks on language, visual attention, spatial neglect, praxis abilities, visual and verbal memory, calculation, number reading and executive functions. Specific tests were also administered to better evaluate different cognitive functions. The Prose Memory Test (immediate and delayed recall) and Interference Memory test [21] were used as a measure of verbal memory. Forward and backward digit span and the Corsi block-tapping test were administered to measure short-term memory and working memory both for the verbal and visuospatial components [22]. Selective attention and switching abilities were measured using the Trail-Making-Test, forms A and B [21]. Different components of language abilities were assessed through specific tests: Phonemic Fluency test [21], the Boston Naming Test for visual naming ability [23], verbal comprehension of words and sentences and repetition of words and non-words [24]. Concerning language domain, the baseline preoperative assessment showed an impaired performance in naming and verbal fluency, whereas the other language abilities were normal (Table 1). Furthermore, the patient refused the proposition of an awake surgery. Therefore, we decided to test the patient for both languages by means of rnTMS integrated with DTI-FT. Due to her anxious state only the dominant hemisphere was evaluated, focused on the surgical planning.

### 2.3. Patient’s Informed Consent

The patient signed specific informed consent for MRI acquisition, rnTMS tests, neuropsychological evaluation and surgical intervention. 

### 2.4. MRI Acquisition

The patient underwent brain MRI according to a specific protocol designed for the nTMS and DTI-FT using a 3T scanner (Ingenia 3T, Philips Healthcare) to obtain 3D T1-weighted images (TR/repetition time = 8, TE/echo time = 3.7); 3D FLAIR/fluid attenuated inversion recovery (TR = 4800, TE = 299, TI/inversion time = 1650, flip angle = 40, matrix = 240 × 240 mm^2^, voxel = 1 × 1 × 1 mm^3^, 196 slices, 4.05 min of acquisition time); diffusion weighted sequences (DWI with 32 directions, TR = 8736, TE = 91; single shell, b = 800 s/mm^2^) for DTI-FT.

### 2.5. nTMS Language Cortical Mapping and Off-Line Analysis

The 3D T1-weighted sequence was imported into the nTMS system (NBS system 4.3—Nexstim Oy, Elimäenkatu 9 B, Helsinki, Finland) for language mapping, performed thorough a repetitive stimulation (rnTMS) according to the most update indications [25,26]. The patient’s resting motor threshold (RMT) was determined by applying nTMS to the left motor cortex representing the hand, detecting the motor response of the m. abductor pollicis brevis. The patient performed the language assessment (base-line test, rnTMS mapping) first in Romanian (in the presence of an interpreter) and then in Italian. The base-line test was performed twice without stimulation, in order to cross out from the list the unfamiliar words, possible confounding variables in error analysis. A total of 80 black-and-white drawings of high and low frequency objects were presented on a 17-inch monitor placed 1 m in front of the patient for the picture naming task. Display and inter-picture time were set at 700 ms and 2500 ms, further adjusted to 2 s and 4 s for both languages. The patient was asked to say aloud the initial phrase “this is a…” to distinguish between a speech arrest and anomia [27]. At the end of the base-line test, 70 and 67 figures were considered for Romanian and Italian mapping, respectively. The rnTMS stimulation frequency was set at the beginning at 5 pulses at 5 Hz at 110% RMT and then increased to 10 pulses at 10 Hz at 100% RMT because with the previous parameters of stimulation we did not obtain any error. The stimulation coil was randomly moved between the presentation of the images in about 1-cm steps over the perisylvian and peritumoral cortex. The rnTMS pulse train automatically triggered with picture presentation (0 ms) [26]. The entire mapping session was recorded on video for off-line data analysis, performed by an expert neuropsychologist (S.F.), helped by an interpreter for the review of the test performed in Romanian. The errors were classified according to Corina et al.: semantic paraphasias, circumlocutions, phonological paraphasias, neologisms, performance errors and no response errors [28]. We considered a site as language-eloquent if at least two of three stimulations caused an error response [25]. The stimulation sessions were well tolerated with a minimal discomfort reported (Visual Analogue Scale 2/10).

**Figure 2:**
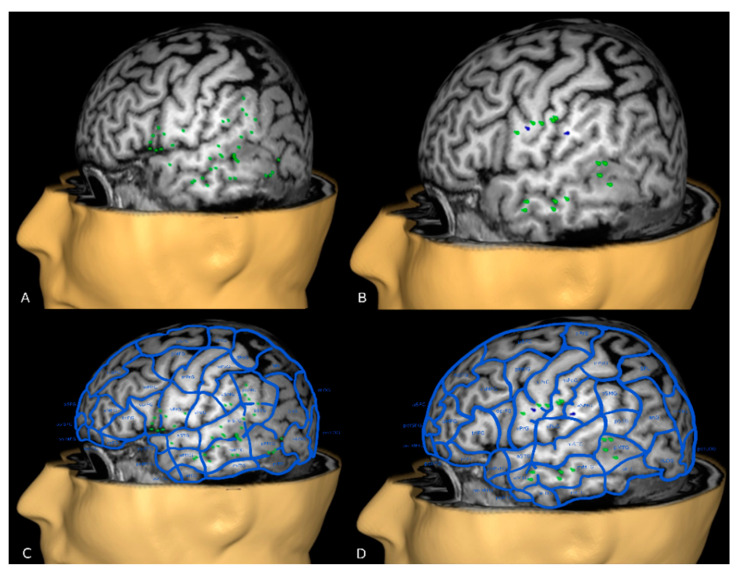
(**A**) Romanian rnTMS mapping (green spots: performance errors) and (**B**) Italian rnTMS mapping (green spots: performance errors, blue spots: semantic errors). (**C**,**D**) show the anatomical distribution of all errors according to the parcellization system area described by Corina et al., in Romanian and Italian, respectively.

The off-line analysis highlighted 39 performance errors in Romanian (320 spots tested) of which a group of 5 was located in the superior–anterior and posterior–inferior border of the lesion. In Italian, 2 semantic and 15 performance errors were detected (271 sites tested), 3 of them located in the anterior part of the tumor. The language maps showed a convergence of the errors in the anterior middle temporal gyrus, middle middle temporal gyrus, posterior middle temporal gyrus, ventral precentral gyrus and anterior supramarginal gyrus according to the cortical parcellation system as described in Corina et al. [29] (Figure 2). The latest convergence corresponds to the anterior part of the tumor. 

### 2.6. nTMS Based DTI-FT of Language Pathway

**Figure 3:**
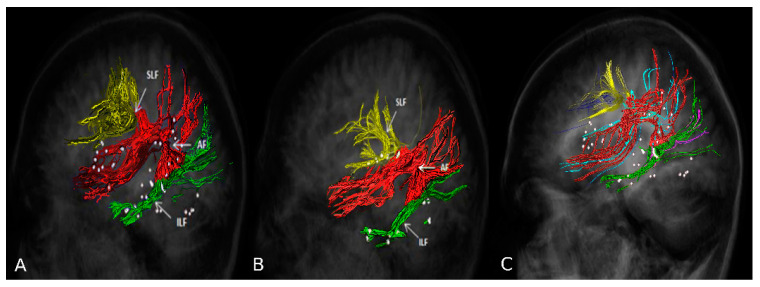
nTMS-based DTI-FT reconstructions of the language subcortical network in Romanian (**A**) and Italian (**B**) identifies the arcuate fascicle (AF, red), the superior longitudinal fascicle (SLF, yellow) and the inferior longitudinal fascicle (ILF, green) for both languages. In (**C**) the overlap of subcortical tracts is depicted. Italian color code—AF: red; SLF: yellow, ILF: green; Romanian color code—AF: light blue, SLF: blue, ILF violet.

The rnTMS cortical mapping was used to obtain the nTMS-based DTI-FT of the principal subcortical pathways of language function: arcuate fascicle (AF), frontal aslant tract (FAT), inferior fronto–occipital fascicle (IFOF), inferior longitudinal fascicle (ILF), superior longitudinal fascicle (SLF), uncinate fascicle (UF) [13,30,31]. The workflow for DTI-FT was performed on the StealthStation S7 navigation system by using StealthViz software (Medtronic Navigation, Coal Creek Circle Louisville, CO, USA). A deterministic approach based on the fiber assignment by continuous tracking (FACT) algorithm was used, with these parameters: FA cut off value = 0.15; vector step length = 0.5 mm; minimum fiber length = 30 mm; seeding density = 1.0; max directional change 90°. All language positive spots were imported into the planning station and used to create an overall object with an additional 5-mm border for each cortical spot. Subsequently, the object was exploited like a single ROI for tracking and the StealthViz software created a directionally encoded color map and then a 3D volume of white matter fibers originating from the cortical positive spots previously selected [11]. nTMS-based DTI-FT was able to identify the subcortical network for both languages, consisting of 533 and 293 fibers for Romanian and Italian, respectively. The 3D volumes were then manually elaborated to better visualize the single language-related tracts included in the reconstruction (i.e AF, SLF and ILF) under constant supervision of an expert neuroradiolgist [32,33,34] (Figure 3). White matter reconstruction displayed an overlap of AF in both languages with the anterior part of the lesion. 

### 2.7. Presurgical Planning

**Figure 4:**
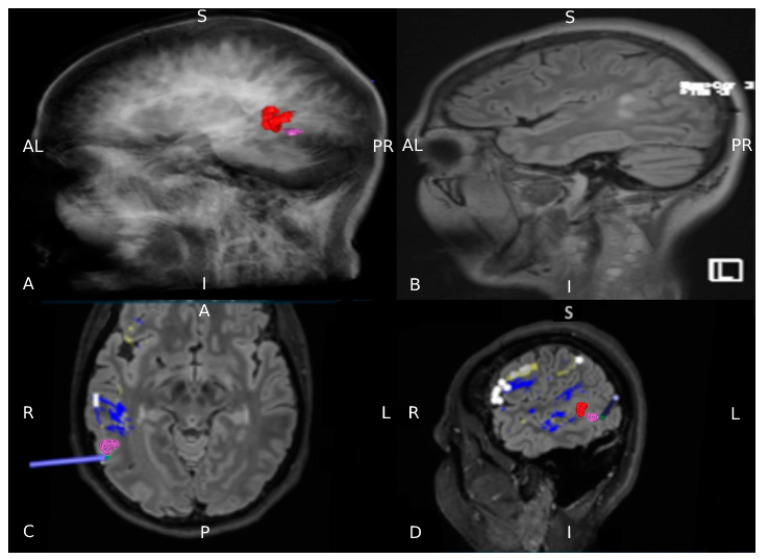
rnTMS-based planning using StealthViz software. (**A**) The anterior and eloquent part of the lesion identified by nTMS is colored in red and the posterior non-eloquent part in violet, (**B**) 3D FLAIR anatomical images for comparison. (**C**,**D**) A subtotal resection was planned and guided by neuronavigation (in violet the non-eloquent part).

According to the rnTMS results, the tumor was divided into an eloquent and non-eloquent part, the latter identified as our surgical target. Using the planning station, the anterior part was highlighted in red and the target in violet. Then, the final reconstruction of language network was imported into the neuronavigation system to assist surgery (Figure 4).

### 2.8. Surgical Intervention and Neuropsychological Follow Up

Surgical resection of the posterior non-eloquent part was achieved by neuronavigation because the lesion was not clearly distinguished from normal brain parenchyma. Integrated histological and molecular diagnosis disclosed a WHO-grade IV gliomas [35]. She received perioperative antiepileptic drugs prophylaxis. Moreover, the patient received dexamethasone 4 mg four times daily for one week followed by gradual tapering. Postoperative neuropsychological assessment, performed after one week, showed a global worsening of the performance in language tasks (reading, number writing) and in other cognitive functions (praxical function in right hand, short- and long-term verbal memory, verbal working memory). However, the follow-up evaluations performed at 1 and 4 months after surgery, revealed a restoration of functions through the time. The performance at four months after the surgery was comparable with the baseline (Table 1). Relatives reported intact native language performance as well. The patient underwent whole brain radiotherapy (60 Gy/30 fractions) and medicated with Temozolomide (two cycles). The patient did not present seizures at last follow-up (10 months).

## 3. Discussion

Despite awake surgery associated with DCS still represents the gold standard for language mapping, an accurate preoperative assessment of language pathway is required to establish the best surgical strategy, for the risk–benefit balance and for the patient’s counselling [36,37,38]. This is mandatory especially in case of patients with lesions located in eloquent areas who are not eligible for awake surgery [39]. Commonly, fMRI is the most accessible and applied preoperative mapping technique, providing the identification of eloquent cortical areas for different types of functions. Nevertheless, the indirect signal of area activation provided near a brain lesion could be undermined by a metabolic uncoupling induced by the lesion itself, determining a reduced fMRI signal in perilesional eloquent cortex [40,41,42]. This phenomenon, associated to a normal or increased activity in homologous brain regions, can simulate a reorganization of the function [43]. Moreover, previous studies have not clarified the reliability of fMRI for preoperative language mapping in tumors located in language-eloquent areas [42,44,45] thus, the use of fMRI in adjunct to other mapping methods is suggested [3,6,46].

nTMS is a recent and promising preoperative mapping technique for cortical functions localization and the development of nTMS-based DTI-FT allows a functionally oriented white matter reconstruction. In fact, the white matter reconstruction based on the rnTMS mapping showed a more accurate and reliable reconstruction of the subcortical language pathway compared to the standard anatomical technique [10,13]. Nonetheless, few centers have a broad experience with this technique and the language mapping has been less investigated compared to the motor nTMS mapping [6]. This may reflect the fact that language function is the result of a complex cortical and subcortical network which is more difficult to localize and challenge to map [28,47,48,49]. Currently, rnTMS combined with nTMS-based DTI-FT could be remarkably useful for patients who are not eligible for awake surgery, providing information concerning the true eloquence of the lesion with a high specificity of rnTMS in localizing language-negative areas. Furthermore, this technique can identify the presence of intra-hemispheric tumor-induced plasticity [14] or inter-hemispheric function reorganization and/or migration involving the non-dominant hemisphere [50,51,52].

For bilingual and multilingual neurosurgical patients affected by lesion located in language-eloquent areas, the preoperative mapping and languages preservation represent an additional major goal. At present, bilingual (and multilingual) neurosurgical patients have been investigated mostly with DCS and fMRI, as highlighted in a very recent review by Polczynska and Bookheimer [16]. This review suggests several principles concerning languages organization in bilingual patients, which may be useful in predicting the likelihood of separate versus converging representation of languages (i.e., age of L2 acquisition, proficiency level of L2 and linguistic distance between L1 and L2). Nonetheless, fMRI may falsely identify certain brain regions as potentially eloquent as above mentioned. Moreover, DCS evaluates a restricted coverage within language areas, mostly focused on sites in the frontal or posterior languages eloquent pathway. Unexpectedly, rnTMS has not been applied to study neurosurgical bilingual patients so far and late bilingual population has not been investigated.

We described the case of a bilingual patient affected by brain tumor located in language-eloquent region. The patient refused to undergo an awake craniotomy. Therefore, in order to deal with the aim of a safe resection, we applied an alternative method that could offer an accurate mapping of both languages. Preoperative languages mapping was obtained by rnTMS and nTMS-based DTI-FT language assessment according to the protocol for rnTMS language mapping used at our institution and established in the literature [11,25,53]. Language mapping for Romanian (L1) and Italian (L2) showed a convergence in the posterior areas of language pathways [48]. This overlapping may be explained by the high proficiency of L2 identified by extensive neuropsychological assessment and by the common derivation from Romance language as previously described. Furthermore, the language mapping disclosed the true eloquence of the anterior part of the lesion for both L1 and L2, limiting the surgical target to the non-eloquent region. Despite the worsening of language tasks and in some cognitive functions, the short-term follow-up highlighted a restoring of functions, comparable with the baseline. Probably this transient worsening was imputable to the surgical manipulation of subcortical fibers producing a functional, rather than anatomical damage. When performing language assessment in a bilingual patient, the setting should consider the presence of an interpreter both during rnTMS and off-line analysis, if the L1 is not properly known by one of the clinical staff members. Ideally, the native language version of the neuropsychological tests should be available and administered by a properly educated interpreter, to achieve a greater accuracy. This can be considered as an intrinsic limitation and possible bias when analyzing different languages, but it can be overcome by the advantages of an accurate mapping of the currently speaking languages, which requires to be preserved. Moreover, rnTMS still presents other pitfalls that need to be assessed. In fact, the likelihood of detecting language-positive spots is still low, drawing attention to the necessity of a revision of current stimulation protocols [49]. Furthermore, the interpretation of the hesitation errors varies among authors, constituting a matter of debate [49,54,55] and, in addition, the pre-existing moderate aphasia or severe cognitive impairment could undermine the reliability of the examination, entailing an accurate patients selection [56]. Nonetheless, the use of the initial sentence during picture naming, helping to distinguish between speech arrest and anomia is not routinely applied [25,27]. Another point is the influence of the antiepileptic drugs on the cortical excitability, which may influence the stimulation threshold as reported for the motor cortex but not investigated for extra-motor cortex [57]. Regarding the functional tractography obtained from the language mapping, a meaningful and debatable protocol should be assessed [10,11,13].

## 4. Conclusions

Our experience showed the reliability of rnTMS mapping in a bilingual patient who required surgery for a language-eloquent lesion for both languages. The potentials of this technique are different. First of all, the clinical application in safe neurosurgical practice is clear, because it represents a good tool for pre-surgical mapping, when awake surgery is not applicable for different reasons and rnTMS may allow filling of this gap. Furthermore, in the specific case of brain tumor, the preoperative mapping with nTMS-based DTI allows a better comprehension of language pathway reorganization and plasticity. A second important application concerns the neural basis of language, and bilingualism in particular, which remain still unclear in the literature. In this context, further studies with rnTMS on bilingual patients and healthy subjects are advocated to a comprehensive study of languages organization and plasticity.

## Figures and Tables

**Table 1:** Neuropsychological assessment.

Assessment	Pre-Operative		Post-Operative		Follow-Up 1 Month		Follow-Up 4 Months	
*Test*	*CS*	*Performance*	*CS*	*Performance*	*CS*	*Performance*	*CS*	*Performance*
**GLOBAL COGNITIVE FUNCTIONS**								
Oxford Cognitive Screen (OCS)								
Denomination	3	Impaired	3	Impaired	3	Impaired	4	Normal
Semantics	3	Normal	3	Normal	3	Normal	3	Normal
Orientation	4	Normal	4	Normal	4	Normal	4	Normal
Visual field	4	Normal	4	Normal	4	Normal	4	Normal
Reading	15	Normal	13	Impaired	15	Normal	15	Normal
Number writing	3	Normal	2	Impaired	3	Normal	3	Normal
Calculation	3	Borderline	3	Borderline	4	Normal	3	Normal
Visual search	47	Normal	47	Normal	46	Normal	49	Normal
egocentric neglect	−1	Normal	1	Normal	2	Normal	1	Normal
allocentric neglect	0	Normal	0	Normal	0	Normal	0	Normal
Imitation								
Right hand	11	Normal	8	Impaired	12	Normal	12	Normal
Left hand	12	Normal	12	Normal	12	Normal	12	Normal
Memory								
Verbal	3	Normal	3	Normal	2	Impaired	3	Normal
Episodic	4	Normal	4	Normal	4	Normal	4	Normal
Executive functions	−1	Normal	−2	Normal	0	Normal	0	Normal
**LANGUAGE**								
Boston Naming Test (15 items)	5	Impaired	3	Impaired	6	Impaired	6	Impaired
E.N.P.A.								
Verbal comprehension (words)	18.4	Normal	18.4	Normal	18.4	Normal	20	Normal
Verbal comprehension (sentences)	14	Normal	14	Normal	14	Normal	14	Normal
Repetition (words)	10	Normal	10	Normal	10	Normal	10	Normal
Repetition (nonwords)	5	Normal	5	Normal	5	Normal	5	Normal
Phonemic Fluency (Mondini, 2011) [21]	7.7	Impaired	1.7	Impaired	1.7	Impaired	4.3	Impaired
**ATTENTION**								
Trail Making Test								
A	26″	Normal	37″	Normal	51″	Normal	46″	Normal
B	167″	Impaired	167″	Impaired	156″	Impaired	133″	Normal
**MEMORY**								
Digit span								
Forward	4.75	Normal	2.75	Impaired	4.75	Normal	4.75	Normal
Backward	3.71	Normal	0	Impaired	3.71	Normal	3.79	Normal
Corsi Test								
Forward	6.74	Normal	5.74	Normal	4.74	Normal	3.81	Normal
Backward	5.67	Normal	5.67	Normal	5.67	Normal	3.79	Normal
Prose Memory								
Immediate	9	Normal	5	Impaired	12	Normal	10	Normal
Delayed	12	Normal	NE	Impaired	15	Normal	17	Normal
Memory Interference								
10 s	8	Normal	5	Normal	8	Normal	8	Normal
30 s	7	Normal	6	Normal	7	Normal	8	Normal

TCS: correct score (the raw score is adjusted for age and education basing on Italian-normative data from the literature, when appropriate). E.N.P.A.: Esame neuropsicologico per l’afasia (i.e., neuropsychological examination for aphasia). NE: not executable. The impairment of the performance is defined basing on cut-off, from normative data from the literature.

## Data Availability

The data presented in this study are available on request from the corresponding author.

## Abbreviations

AF: arcuate fascicle; DCS: direct cortical stimulation; DTI-FT: diffusion tensor imaging-fiber tracking; FAT: frontal aslant tract; IFOF: inferior fronto-occipital fascicle; ILF: inferior longitudinal fascicle; fMRI: functional MRI; L1: first language; L2: second language; nTMS: navigated navigate transcranial magnetic stimulation; RMT: resting motor threshold; rTMS: repetitive transcranial magnetic stimulation; rnTMS: repetitive navigate transcranial magnetic stimulation; SLF: superior longitudinal fascicle; UF: uncinate fascicle.
